# Benefits of Peer Support in Online Japanese Breast Cancer Communities: Differences Between Lurkers and Posters

**DOI:** 10.2196/jmir.1696

**Published:** 2011-12-29

**Authors:** Yoko Setoyama, Yoshihiko Yamazaki, Kazuhiro Namayama

**Affiliations:** ^1^Department of Nursing InformaticsSt Luke’s College of NursingTokyoJapan; ^2^The Health Care Science InstituteMinato-ku, TokyoJapan; ^3^Public Health Research FoundationInstitute of Stress ScienceTokyoJapan

**Keywords:** Online support groups, social support, patients, lurkers, breast cancer, mental health, Japan

## Abstract

**Background:**

Web 2.0 has improved interactions among peers on the Internet, especially for the many online patient communities that have emerged over the past decades. Online communities are said to be particularly beneficial peer support resources for patients with breast cancer. However, most studies of online patient communities have focused on those members who post actively (posters), even though there are many members who participate without posting (lurkers). In addition, little attention has been paid to the usage of online communities among non-English-speaking patients.

**Objective:**

The present study explored the differences in peer support received by lurkers and posters in online breast cancer communities. It also examined the effects of such support on both groups’ mental health.

**Methods:**

We conducted an exploratory, descriptive, cross-sectional, Web-based survey among members of four Japanese online breast cancer communities. In an online questionnaire, we asked questions regarding sociodemographics, disease-related characteristics, mental health, participation in online communities, and peer support received from those communities.

**Results:**

Of the 465 people who accessed the questionnaire, 253 completed it. Of the respondents, 113/220 (51.4%) were lurkers. There was no significant difference between lurkers and posters with regard to sociodemographic variables. About half of the posters had been given a diagnosis of breast cancer less than a year previously, which was a significantly shorter period than that of the lurkers (*P* = .02). The 5 support functions extracted by factor analysis were the same for both posters and lurkers. These were emotional support/helper therapy, emotional expression, conflict, advice, and insight/universality. When the support scores were calculated, insight/universality scored highest for both posters and lurkers, with scores that were not significantly different between the two groups. Among the 5 support scores, emotional support/helper therapy and emotional expression were significantly higher among posters. For posters, emotional support/helper therapy and advice were negatively correlated with the anxiety subscale of the Hospital Anxiety and Depression Scale. Emotional expression, advice, and insight/universality were negatively correlated with the anxiety subscale for lurkers.

**Conclusion:**

We found that posters felt they received more benefits from online communities than lurkers did, including emotional support, helping other patients, and expressing their emotions. Yet even lurkers were found to gain a certain amount of peer support through online communities, especially with regard to advice and insight/universality. The results demonstrate that participation in online communities—even as a lurker—may be beneficial to breast cancer patients’ mental health.

## Introduction

The Internet has become increasingly popular in Japan since the 1990s. The Internet penetration rate in Japan exceeded 75.3% in 2008 [1], and many Japanese people now use the Internet in their daily lives. After the mid 2000s, people began to interact with each other on the Internet using Web 2.0 functions such as blogs, social networking services, and Q&A websites. Web 2.0 is a term that O’Reilly defined as “a set of economic, social, and technology trends that collectively form the basis for the next generation of the Internet, a more mature, distinctive medium characterized by user participation, openness, and network effects” [2]. Users are now able to post comments freely on the Internet without possessing complex technical skills. Hansen [3] stated that Web 2.0 “improved communication and collaboration between people.” Specifically, one of the outcomes of the popularization of Web 2.0 was that people with similar health experiences developed online support communities [4]. On the basis of this standpoint, it is important to determine what people do and how they communicate with each other via the Internet over the course of their illness.

Online communities are beneficial because of their availability; for instance, they have no time restrictions [5] and people can access them from any region at no cost [6]. Thus, it is easy for people with disabilities and psychological burdens to receive support from peers online. Previous studies have shown that patients with heart disease [7] and other rare diseases [8] shared their experiences and exchanged emotional and informational support through online communities. In addition, Hill and Weinert [9] found that online communities help participants learn more about themselves, overcome isolation, and find companionship while adapting to their illness. Thus, online communities are now considered a beneficial peer support resource for patients [10].

Since there are many treatment options for breast cancer, patients’ informational needs are high. In fact, breast cancer is the most common health topic researched on the Internet. Davison et al [11] reported that support related to breast cancer was the most searched-for health topic on the Internet in the United States, followed by acquired immunodeficiency syndrome, alcoholism, and prostate cancer. Sharf [5] observed that patients with breast cancer exchanged information, social support, and even personal empowerment through online communities. Radin [12] found that breast cancer patients promoted “thick trust” and “collected intelligence” through online communities, and that they discussed various typically painful cancer-related topics with candor, warmth, and even humor [13]. Moreover, online breast cancer communities have been shown to be a useful resource in reducing depression [14,15], dealing with cancer-related trauma [16], and improving posttraumatic growth and psychosocial well-being [14]. Online communities have been found to be comparable in effectiveness with face-to-face support groups [16].

People can participate in online communities in two ways. Those who participate actively are known as posters, and those who do so passively, without making any postings, are known as lurkers [17]. van Uden-Kraan et al [18] found that both posters and lurkers are in some way empowered by participating in online communities; they considered this participation to be a form of bibliotherapy.

Previous researchers have identified some of the reasons why people do not post in online communities, including lack software skills, dislike of the group dynamic, or feeling that the community is a poor fit for them [19]. On the other hand, some people simply do not feel the need to post and feel that they are being helpful by not posting [19]. Many previous studies that describe the benefits of online communities have focused on members who actively contributed by posting messages (ie, posters) [18]. However, Nonnecke and Preece [17] reported that in health-related online communities, an average of 45.5% of people participated as lurkers. If online communities are a peer support resource from which even lurkers can gain some benefit, people who feel that it is a technological and psychological burden to post can use these resources more freely.

To provide further evidence of online communities as a health resource, their effects on users’ health should be explored for both posters and lurkers. Moreover, although the Internet penetration rate in Japan is comparable with that of Western countries [20], there have been limited studies of online communities in Japan. Studies of non-English-language online communities are also scarce [21]. Thus, in the present study, we investigated Japanese online breast cancer support communities to determine whether peer support is received differently by lurkers and posters. In addition, we explored the effects of support on members’ mental health between the two groups.

## Methods

### Survey Procedure

In this exploratory, descriptive, cross-sectional study, we conducted a Web survey from September to October 2007, referring to the checklist for the quality improvement of Web surveys [22].

We searched for online communities designed for breast cancer patients using the Google Japan and Yahoo! Japan search engines, which have the largest and second largest numbers of users in Japan, respectively [23]. When searching for online communities, we used the keywords *breast cancer*, *discussion board*, and *mailing list*. Discussion boards and mailing lists are differentiated by registration systems. However, because our research focus was peer support received by members of Internet communities, the registration system distinctions were irrelevant.

We found 12 different breast cancer communities and asked their administrators for survey cooperation via email. During this process, we eliminated those online communities that had participants with non-breast cancers and those in which health care providers served as managers. All of the participating online communities had new posts within 28 days from the start of the survey. Finally, administrators from 4 of the initial 12 online breast cancer communities agreed to cooperate with this survey. The purpose of all of the communities was the exchange of peer support among breast cancer patients.

We developed an online questionnaire form for this open survey. We did not offer any incentive to participate. The four administrators explained the research to their communities’ members and provided the questionnaire URL by posting information on their respective community websites. The explanation of the research included a statement about the purpose of this study, the survey duration, and how to store the data on a secure server. We used secure websites to protect personal data. The usability and technical functions of the site were tested by a group of colleagues before we conducted the real test. The 5-page survey site had an average of 8 items on each page of the questionnaire.

Participants were able to navigate to the questionnaire site directly from the community sites by clicking on a hyperlink, and we explained that accessing the questionnaire site would be regarded as an agreement to participate in the survey. To prevent multiple entries from the same individuals, we checked the IP address of everyone who participated in the survey.

### Instrument

We did not have a valid instrument to precisely measure social support from peers for posters and lurkers, so we developed a new instrument for the purpose of our study. Of course, there are existing instruments that can be used to measure general social support, such as informational support and emotional support [24], or support networks, such as family members and friends [25]. However, after conducting interviews with seven patients in online breast cancer communities regarding how they used those communities and what kind of support they received from them, we decided to develop a new instrument that could measure social support given specifically by online peers. We then interviewed two nurses in order to check the face validity of our instrument. These nurses were specialists in breast cancer care.

#### Sociodemographic Characteristics

Our survey inquired about patients’ age, marital status (unmarried, married, or separated/widowed), education (middle school, high school, vocational school/2-year college, university/graduate school or higher), and employment (full-time job, housewife, part-time job, or unemployed). All of the participants were women.

#### Disease-Related Characteristics

The respondents were asked to report on four disease-related characteristics: (1) time since diagnosis of breast cancer (less than 1 year, 1–2 years, 3–5 years, 6–9 years, and 10 years or more, (2) stage of breast cancer at the time of diagnosis (below stage I to beyond stage III), (3) physical symptoms due to breast cancer or breast cancer treatment (eg, pain, feeling tired, arm paralysis, and nausea—respondents who selected more than 1 symptom were categorized as patients with symptoms, and we also counted the total number of symptoms), and (4) personal daily activity level, indicating physical condition. Activity level was indicated using a 5-point Likert scale that ranged from 5, living completely as usual, to 1, almost staying in bed.

#### Mental Health

Patients rated their levels of anxiety and depression on the Hospital Anxiety and Depression Scale (HADS), which has been used with the general population, cancer patients, and primary care patients [26]. The HADS consists of 14 items: 7 on the depression subscale and 7 on the anxiety subscale. Each item is scored on a 4-point scale from 0 (not present) to 3 (considerable), and the item scores are added, yielding anxiety and depression scores from 0 (minimum symptom load) to 21 (maximum symptom load). A higher score indicates a worse condition. A Japanese version of the scale has been widely used and was confirmed to be reliable and valid [27]. Cronbach alpha for the total HADS score in this study was .89.

#### Participation in Online Communities

We asked the participants “How often do you post in online communities?” The response items were every time, sometimes, or never—just lurking. We labeled respondents who selected every time and sometimes as posters and those who selected never—just lurking as lurkers.

#### Received Peer Support

On the basis of our previous interviews, we extracted 8 categories of peer support that study participants received by taking part in online communities. These categories were emotional support, informational support/advice, insight, emotional expression, universality, conflict, empowerment, and helper therapy. Emotional support and informational support were the functions of social support that Cohen et al found in their studies [24]. Insight, universality, modeling, and helper therapy correspond to the concepts that Mishima et al [28], Takahashi et al [29], and Hirose et al [30] found to be the functions of self-help groups. Empowerment corresponds to the study of van Uden-Kraan et al [18]. Conflict has been found to correspond to negative experiences when patients participate in face-to-face support groups [31].

On the basis of these concepts, we formulated 34 items that described the peer support that took place in the online communities. All items had the format of a statement that began with the phrase “Through my participation in online communities...” Respondents could answer on a 5-point Likert scale that ranged from 5 (strongly agree) to 1 (strongly disagree). Emotional support, informational support/advice, insight, and universality were measured with 4 items; conflict was measured with 7 items; empowerment was measured with 4 items; and helper therapy was measured with 3 items.

### Analysis

The incidence and average scores of the sociodemographic variables and the current status of participation were calculated for posters and lurkers. Metric variables were analyzed by *t* tests, and categorical variables were analyzed with chi-square tests and Kruskal-Wallis tests. For 61 patients with breast cancer who used online communities, we conducted a pilot test in order to check whether the quantity and quality of the questionnaire was suited to our study’s objectives. Then, we revised some of the words to which the patients said they could not respond very well. We also deleted 2 items from insight, 1 item from universality, and 2 items from empowerment because of the floor and ceiling effects. We thereby used only 29 items to measure peer support received from online communities. We did not include the data of the pilot test samples in the final analysis.

 We conducted an exploratory factor analysis to evaluate the factor structure of the support functions for posters and lurkers. While we knew the expected factors based on the previous research used to construct the items, we chose an exploratory factor analysis to determine the best factors for these data. We used principal axis factoring with promax rotation, an oblique rotation method that minimizes the number of variables with high loadings on each factor. This method simplifies the interpretation of the factors. We specified a precedent cut-off of .35 for acceptable factor loadings. To compare the factor constructions between posters and lurkers, we conducted a separate factor analysis for the extracted factors.

After conducting a factor analysis, we deleted 2 items from empowerment, 2 from helper therapy, and 1 from universality because the factor loadings of these items were all less than .35. Considering the factor loadings of each item and the content validity, we extracted 5 factors from the instrument. We then calculated the sum of the scores for each support function, which we referred to as the support score. To compare support scores between posters and lurkers, we conducted an analysis of variance (ANOVA) using a general linear model, controlling for time since diagnosis. We then calculated the Pearson correlation coefficient to determine the relationship between each health status (HADS) and support scores.

### Ethical Consideration

We explained the aim of the research project both verbally and in writing to the administrators of the online communities. They were assured that anonymity would be guaranteed and that refusing to participate or withdrawing consent would have no negative consequences. Since the investigation of patients may lead to psychological stress, we made special efforts to reduce the psychological burden of the questionnaire survey and exercised the utmost caution to protect participants’ privacy. The Ethics Review Committee of the University of Tokyo approved this study (approval number: 1789).

## Results

### Participants’ Characteristics

The number of visitors to the questionnaire site, or unique site visitors, was 465. We clarified the number of unique visitors based on IP addresses. The number of people who completed the questionnaire was 253. The completion rate, or the ratio of people who agreed to participate to the number of those who finished the survey, was 0.544.

To ensure valid data from a homogeneous sample, we excluded 33 participants: those who had recurrent breast cancer (n = 21), those who had not undergone any surgery for breast cancer (n = 8), and those who had an extremely low daily activity level (“almost staying in bed”) (n = 4). Ultimately, we analyzed 220 valid responses. We only analyzed completed questionnaires. The average time in which participants answered the questionnaire was 27 minutes. There were no outliers.

The respondents’ active participation in online communities was as follows: every time, n = 14 (6.4%); sometimes, n = 93 (42.2%); and never—just lurking, n = 113 (51.4%).

The characteristics of the survey respondents are shown in Table 1 and Table 2. No variables differed significantly between posters and lurkers. About half of the posters had their breast cancer diagnosis within the previous year, a period that was significantly shorter than that of lurkers (*P* = .02).

**Table 1:** Sociodemographic characteristics of posters and lurkers (n = 220) (excluding missing data)

		Posters (n = 107)		Lurkers (n = 113)		*P* value
		n	%	n	%	
**Age (years)**						.55^a^
	≤29	2	2	2	2	
	30–39	24	23	30	27	
	40–49	60	58	55	50	
	50–59	16	15	22	20	
	60–69	2	2	2	2	
	Mean (SD)	43.71 (7.197)		44.79 (7.474)		.66^b^
**Marital status**						.24^a^
	Unmarried	16	16	30	28	
	Married	77	75	62	57	
	Separated/widowed	10	10	16	15	
**Education**						.13^a^
	High school	22	21	31	29	
	Vocational school/2-year college	34	33	43	40	
	University/graduate or higher	47	46	34	31	
**Employment**						.89^a^
	Full-time	30	28	33	30	
	Housewife	37	35	32	29	
	Part-time	22	21	30	27	
	Unemployed	18	17	17	15	

^a^ χ^2^ test. Degrees of freedom were the number of category –1.

^b^ *t* test. Degree of freedom was 219.

**Table 2:** Health characteristics of posters and lurkers (n = 220) (excluding missing data)

		Posters (n = 107)		Lurkers (n = 113)		*P* value
		n	%	n	%	
**Time since diagnosis (years)**						.02^a^
	<1	52	49	31	38	
	1–2	33	31	39	35	
	3–5	9	8	23	21	
	6–9	10	9	9	8	
	≥10	2	2	8	7	
**Cancer stage at diagnosis**						.39^b^
	I	50	47	36	34	
	II	43	41	48	45	
	III+	8	8	13	12	
	Not known	5	5	9	8	
**Presence of symptoms^c^**						.26^b^
	Yes	93	87	85	75	
	No	14	13	28	25	
Number of symptoms, mean (SD)		2 (1.685)		2 (1.456)		.62^d^
**Physical condition**						.77^a^
	Living completely as usual	57	53	58	51	
	Living as usual	50	47	55	49	
**HADS^e^, mean (SD)**						
	Summed scores	12.6 (6.9)		13.4 (8.7)		.52^d^
	Depression	6.2 (3.6)		6.5 (4.1)		.63^d^
	Anxiety	6.4 (4.1)		6.9 (5.4)		.51^d^

^a^ Kruskal-Wallis test.

^b^ χ^2^ test. Degrees of freedom were the number of category –1.

^c^ Respondents checked all of their current symptoms due to breast cancer (eg, pain, tiredness, paralysis of arm, and nausea) and were classified as having symptoms if they chose more than 1 symptom.

^d^ *t* test. Degree of freedom was 219.

^e^ Hospital Anxiety and Depression Scale.

### Support Functions From Online Communities for Posters and Lurkers

The 5 peer support factors that we extracted from the poster and lurker groups were the same (Table 3, Table 4). These 5 factors, which each group felt that they received from peers in their online community, were emotional support/helper therapy, emotional expression, conflict, advice, and insight/universality. Each factor had a Cronbach alpha > .65.

**Table 3:** Factor analysis of peer support functions for posters (n = 107)

Factor (Cronbach alpha)		Factor loading extracted for each factor
**Emotional support/helper therapy (alpha = .752)**		
	I was encouraged when I was supported by peers	.777
	I began to respond positively to my peers	.767
	I could talk pleasantly with my peers about topics besides breast cancer	.732
	I was encouraged when I could help my peers	.644
	I wanted to be as cheerful as my happier peers	.613
	I wanted to help other patients who were troubled with breast cancer	.574
	I wanted to make others aware of breast cancer	.476
**Emotional expression (alpha = .850)**		
	I could straightforwardly express my feelings about relationships in my workplace or family	.848
	I could express my feelings about my relationship with my own doctor	.819
	I could straightforwardly talk about my condition	.703
	I could express my feelings after breast cancer diagnosis	.518
**Advice (alpha = .739)**		
	I received advice about treatment decision making and the side effects of various treatments	.725
	I received advice about day-to-day life with breast cancer, such as a wig and mastectomy bra	.672
	I received advice about relationships with family members or colleagues in my workplace	.520
	I received advice about my relationship with my doctor and about selecting a hospital	.505
**Conflict (alpha = .652)**		
	I could not express my feelings out of consideration for others	.605
	I was concerned that I might get incorrect information about breast cancer	.580
	I became tired when breast cancer became the only topic of conversation	.506
	I felt discomfort when I was misunderstood by my peers	.497
	I regretted that I learned about a better treatment from peers after finishing my treatment	.484
	I felt burdened by the time and cost of the peer support resource	.463
	I was in trouble when peers recommended I buy some useless products	.383
**Insight/universality (alpha = .674)**		
	I could help myself recover after I realized that my experience was not unique	.688
	I had more insight about myself after meeting other patients	.580
	I calmed down when I met other patients who had similar experiences to mine	.573

**Table 4:** Factor analysis of peer support functions for lurkers (n = 113)

Factor (Cronbach alpha)		Factor loading extracted for each factor
**Emotional support/helper therapy (alpha = .786)**		
	I was encouraged when I was supported by peers	.505
	I began to respond positively to my peers	.547
	I could talk pleasantly with my peers about topics besides breast cancer	.703
	I was encouraged when I could help my peers	.738
	I wanted to be as cheerful as my happier peers	.573
	I wanted to help other patients who were troubled with breast cancer	.814
	I wanted to make others aware of breast cancer	.956
**Emotional expression (alpha = .910)**		
	I could straightforwardly express my feelings about relationships in my workplace or family	.911
	I could express my feelings about my relationship with my own doctor	.839
	I could straightforwardly talk about my condition	.974
	I could express my feelings after breast cancer diagnosis	.925
**Advice (alpha = .808)**		
	I received advice about treatment decision making and the side effects of various treatments	.642
	I received advice about day-to-day life with breast cancer, such as a wig and mastectomy bra	.873
	I received advice about relationships with family members or colleagues in my workplace	.671
	I received advice about my relationship with my doctor and about selecting a hospital	.854
**Conflict (alpha = .796)**		
	I could not express my feelings out of consideration for others	.554
	I was concerned that I might get incorrect information about breast cancer	.619
	I became tired when breast cancer became the only topic of conversation	.747
	I felt discomfort when I was misunderstood by my peers	.767
	I regretted that I learned about a better treatment from peers after finishing my treatment	.460
	I felt burdened by the time and cost of the peer support resource	.652
	I was in trouble when peers recommended I buy some useless products	.735
**Insight/universality (alpha = .822)**		
	I could help myself recover after I realized that my experience was not unique	.926
	I had more insight about myself after meeting other patients	.627
	I calmed down when I met other patients who had similar experiences to mine	.899

### Support Scores of Posters and Lurkers

**Figure 1:**
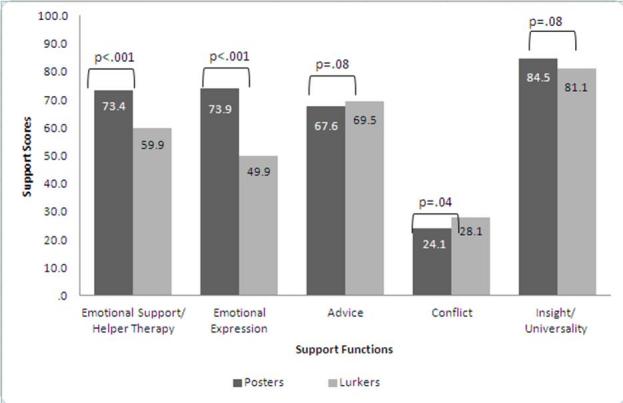
Support scores for posters and lurkers.

Each support score, determined based on the extracted factors, is shown in Figure 1. All scores were converted to be out of 100 points. The highest score was for insight/universality for both posters and lurkers. In the results of ANOVA using the general linear model, controlled by time since diagnosis, there was no significant difference between these scores (*P* = .08). The scores for emotional support/helper therapy (*P* < .001) and emotional expression (*P* < .001) were significantly higher for posters.

### Correlation Between Support Functions and Mental Health

We calculated the correlation between each support and mental health score (HADS) for both posters and lurkers, as shown in Table 5.

For posters, emotional support/helper therapy (*r* = –.477, *P* < .001) and advice (*r* = –.399, *P* < .001) were negatively correlated with the anxiety subscale. Conflict (*r* = .287, *P* = .001) was positively correlated with the depression subscale. For lurkers, emotional expression (*r* = –.294, *P* < .001), advice (*r* = –.655, *P* < .001), and insight/universality (*r* = –.495, *P* < .001) were negatively correlated with the anxiety subscale. Emotional expression (*r* = –.116, *P* = .05) also had a slightly negative correlation with the depression subscale.

**Table 5:** Correlations between support score and mental health as measured by the Hospital Anxiety and Depression Scale (HADS) subscales anxiety and depression (n = 220) (excluding missing data)

		Anxiety		Depression	
		*r*	*P* value	*r*	*P* value
**Posters (n = 107)**					
	Emotional support/helper therapy	–.477	<.001	.002	.99
	Emotional expression	.090	.30	.045	.60
	Advice	–.399	<.001	.082	.34
	Conflict	.132	.12	.287	.001
	Insight/universality	.130	.13	–.007	.93
**Lurkers (n = 113)**					
	Emotional support/helper therapy	.042	.47	.048	.41
	Emotional expression	–.294	<.001	–.116	.05
	Advice	–.655	<.001	.004	.95
	Conflict	.049	.40	.093	.11
	Insight/universality	–.495	<.001	–.048	.41

## Discussion

Most of the posters who participated in our study had received a breast cancer diagnosis relatively recently. Notably, this result does not match that of the study of van Uden-Kraan et al [18]. The participants in our survey were all patients with breast cancer, which was different from the samples of previous studies that included patients with chronic disease. Patients with breast cancer are faced with major decisions about their treatment during a short period just after their diagnosis. Thus, in the process between diagnosis and decision making, their informational needs are high and they experience psychological distress [32]. The participants in this study may also have had high informational needs before making major decisions or just after beginning their treatment. Thus, it is possible that patients who have a recent diagnosis may use online communities actively as posters in order to ask questions and express their emotions.

Also in contrast to the study of van Uden-Kraan et al [18], in our study we did not find a significant age difference between posters and lurkers. They attributed this difference to the lack of computer skills of older people. However, in Japan, the penetration of the Internet among people 60 years or older has grown rapidly, from 37.6% in 2008 to 58.0% in 2009, so their familiarity with computers has increased [23]. Thus, in Japan, the difference between posters and lurkers is not thought to be influenced by a lack of computer skills resulting from age. Instead, these differences could be influenced by the level of people’s informational needs, as mentioned above.

In this study, among the 5 functions of peer support from online communities, emotional support and emotional expression were similar to the peer support provided by face-to-face support groups [24]. These were also defined as group cohesiveness and catharsis in online communities [33]. Goodman [31] defined advice, insight, and universality as peer support functions, while Mishima et al [28] and Vilhauer [33] referred to helper therapy as altruism [33]. Moreover, according to Goodman, conflict is considered to be a negative form of support from peers [31].

To put it simply, we ascertained that the 5 support functions found by this survey characterized social support from peers. Additionally, both posters and lurkers were found to receive some amount of support. Social support plays an important role as a buffer for stressful events such as the diagnosis of a life-threatening disease [34]. Online communities are not just convenient for participants because they are accessible 24 hours a day from anywhere; they also act as a beneficial social support resource, even through passive participation (ie, not posting).

Among the 5 functions, insight/universality scored the highest among both posters and lurkers. Therefore, it can be said that the main function of online communities is to provide insight and universality. In our study, scores for emotional support/helper therapy and emotional expression differed significantly between posters and lurkers. So emotional support/helper therapy and emotional expression may be considered to be support that can be received by actively participating in online communities. However, lurkers received a certain amount of these support functions. It can thereby be said that lurkers can feel comforted by online communities, and that they express their emotions without posting because of the modeling effect. People can identify with others more easily by reading or hearing about experiences that are similar to their own [35]; as van Uden-Kraan et al stated [18], lurking in online communities might be seen as a form of bibliotherapy. In addition, lurkers and posters did not have significantly different scores for advice or insight/universality. These results indicate that lurkers, who participate passively, can receive a similar amount of support to that received by posters through active participation.

In this study, the more posters felt they received emotional support/helper therapy and advice, the less anxious they felt. Furthermore, the more advice lurkers gained from their peers, the less anxious they felt. Learning from others who have had similar experiences helps people control their emotions by reducing the number of future unknowns [36-38]. Because our study was cross-sectional, we cannot explain the causal relationship between them. However, theoretically, social support has a positive influence on people’s mental health. Therefore, these associations between received peer support and better mental health may imply that participants reduce their emotional conflict through peer support from online communities. As for advice, people who receive informational support can experience reduced future uncertainty, which can assuage their anxiety. Posters are considered to actively give and receive support, and their actions can positively affect their emotional status. Lurkers can be said to have simulated experiences through reading others’ exchanges in posts.

In our study, the more emotional expression lurkers—who do not express their experiences and feelings directly—received, the less anxious they felt. Iwamitsu et al [39] state that expressing negative and positive emotions appropriately could be beneficial for reducing emotional distress among breast cancer patients. Therefore, our study may partially support his opinion. We found associations between more emotional expression and less anxiety only among lurkers because the lurkers probably read the contents of the online community more carefully than the posters did. It may be easier for lurkers to gain more social modeling effects than for posters, who may not read others’ posts and only post to meet their own needs. Additionally, Silverberg [40] explained the process of bibliotherapy as knowledge about others’ experiences leading to positive outcomes through the mechanism of changes such as insight and catharsis. According to our results, the main function of online communities is to provide insight and universality. Being part of an online community could thereby have a positive effect on mental health. Previously, it was thought that only active participation in online support groups could have a positive effect on mental health [15]. However, this study reveals that online communities may have positive effects for even passive participants as well.

The age group with the most frequent occurrence of breast cancer is women in their 50s [41]. Thus, many patients play multiple roles in their families and careers. It is therefore important to let them know which social support resources can be used with few limitations in terms of time, location, and psychological burden. Moreover, it is important to inform them that these resources may be beneficial for their mental health, even for passive users.

### Limitations

In this study, we asked for cooperation from administrators of online communities found using Google and Yahoo! Japan. Thus, the population of the study sample is considered to contain those who were already Internet users and those who were likely to seek peer support. Additionally, we could not analyze the characteristics of those who did not complete the questionnaire or those who stopped participating in an online community before the samples were recruited. This could mean that people who had a negative impression of online communities eliminated themselves from the survey. Thus, the results may be biased to indicate more positive conditions than those that actually exist. In future, we should identify the characteristics of those who stop using online communities and determine what kind of population is best suited to using this support resource.

Due to the cross-sectional nature of this study, we were unable to determine the causal relationship between received support and mental health. Therefore, it is possible that people with less initial anxiety were more likely to receive peer support. Although it is theoretically reasonable to expect that greater support leads to better health, a longitudinal study is needed to confirm such a causal relationship.

Despite these limitations, this study suggests that even lurkers, who participate passively in online communities, can gain peer support through the Internet, and that some peer support may have a positive effect on their mental health. Health care providers should therefore provide information about online communities as a support resource for patients with breast cancer.

## Abbreviations

ANOVA: analysis of variance
HADS: Hospital Anxiety and Depression Scale
